# Holistic Fusion of Fragmented Signal Features for Automatic Modulation Recognition via an Adaptive Topological Network

**DOI:** 10.3390/s26175540

**Published:** 2026-08-31

**Authors:** Xiang Liu, Yachao Li, Qi Wang, Yanhong Guo, Chunyu Zhu, Ning Zhang

**Affiliations:** 1 Hangzhou Research Institute, Xidian University, Hangzhou 311231, China; 24241214894@stu.xidian.edu.cn (X.L.); ycli@mail.xidian.edu.cn (Y.L.); zhuchunyu@xidian.edu.cn (C.Z.); zhangning1@xidian.edu.cn (N.Z.); 2 School of Economics and Management, Dalian University of Technology, Dalian 116024, China; guoyh@dlut.edu.cn

**Keywords:** automatic modulation recognition, low-SNR signal classification, Kolmogorov–Arnold network, adaptive soft-threshold denoising, topology-aware multi-scale fusion

## Abstract

Automatic modulation recognition (AMR) remains challenging under low-signal-to-noise ratio (SNR) conditions, where severe noise can obscure weak modulation-specific waveform patterns. To address this issue, this paper proposes an Adaptive Holistic Fusion Network (AHFN) for robust low-SNR modulation recognition. AHFN first employs a multi-resolution nonlinear fusion module composed of parallel KAN branches with different spline-grid resolutions to extract complementary waveform dynamics from standardized I/Q samples and temporal positional information. An adaptive soft-threshold denoising module then generates node- and channel-specific thresholds to suppress noise-sensitive responses while preserving discriminative modulation cues. Subsequently, a topology-aware multi-scale fusion network performs feature- and structure-adaptive message processing over a fixed temporal graph and combines multi-scale graph representations with Mamba-based long-range sequence modeling. Experiments on RML2016.10A and RML2016.10B show that AHFN consistently improves recognition accuracy over representative baseline methods from −20 dB to 0 dB, with gains ranging from 3.03% to 14.34%. These results demonstrate the effectiveness of multi-resolution nonlinear representation, adaptive denoising, and topology-aware local–global fusion for low-SNR AMR.

## 1. Introduction

With the rapid development of wireless communications, Internet of Things (IoT) networks, and spectrum-aware intelligent systems, wireless electromagnetic environments have become increasingly dynamic, heterogeneous, and interference-prone, placing higher demands on reliable spectrum perception and efficient spectrum utilization [1,2,3,4]. In non-cooperative and spectrum-sharing scenarios, received signals are frequently affected by noise, multipath fading, channel uncertainty, and synchronization mismatch, making automatic modulation recognition (AMR) particularly challenging under low-signal-to-noise ratio (SNR) conditions. Early AMR methods relied mainly on likelihood-based decision rules and handcrafted features, including higher-order statistics, instantaneous amplitude, phase and frequency characteristics, cyclic features, and constellation- or phase-diagram-based descriptors [5,6,7,8]. These methods generally offer relatively low computational complexity and favorable physical interpretability, while phase-related geometric representations can reveal modulation-specific structural characteristics. Recent nonlinear feature-based methods have employed phase-diagram analysis to extract modulation-specific structural descriptors, providing an interpretable approach for digital modulation characterization under noisy conditions [9]. However, their performance often depends on the selected features and may deteriorate with limited observation lengths, carrier-frequency offset, phase rotation, synchronization errors, or severe noise corruption.

Deep learning-based AMR reduces dependence on manual feature engineering by learning discriminative representations directly from raw I/Q samples or derived signal views. CNN-based models efficiently extract local waveform patterns, while recurrent and hybrid architectures improve temporal dependency modeling and have achieved favorable recognition performance on commonly used benchmark datasets; however, convolutional operations may inadequately capture long-range dependencies, and recurrent structures introduce sequential computation [10,11,12,13,14]. Multi-feature fusion, multi-scale networks, lightweight architectures, time–phase modeling, and transformer-based methods further improve feature diversity, scale adaptability, or global dependency modeling, although their performance gains may be accompanied by increased architectural complexity or dependence on carefully designed fusion strategies [15,16,17,18,19,20]. Complex-valued processing, attention-guided denoising, phase transformation, and shrinkage-based mechanisms preserve phase-related information and enhance low-SNR recognition, but some methods rely on predefined transformations or specific denoising assumptions [21,22,23,24,25]. Snoap et al. introduced a capsule-network-based classifier using cyclic-cumulant features [26] and subsequently developed a neural classifier combining custom feature-extraction layers with temporal I/Q data [27]; these approaches demonstrate the value of structured signal features but still depend on explicitly designed feature representations. In contrast, AHFN learns adaptive noise-robust representations directly from standardized I/Q sequences. More recent scale-aware, transfer-learning, multimodal, graph-based, and contrastive methods have reported competitive recognition performance by modeling long-range dependencies or structural relationships, although they may require auxiliary modalities, source-domain knowledge, predefined graph construction, or additional training objectives [28,29,30,31,32,33,34,35]. Direct numerical comparison among these studies remains limited by differences in data partitions, input lengths, channel conditions, and evaluation protocols. Moreover, robustness against stochastic noise does not necessarily imply robustness against deliberately optimized adversarial perturbations, which represents a distinct vulnerability of deep modulation classifiers [36].

**Figure 1:**
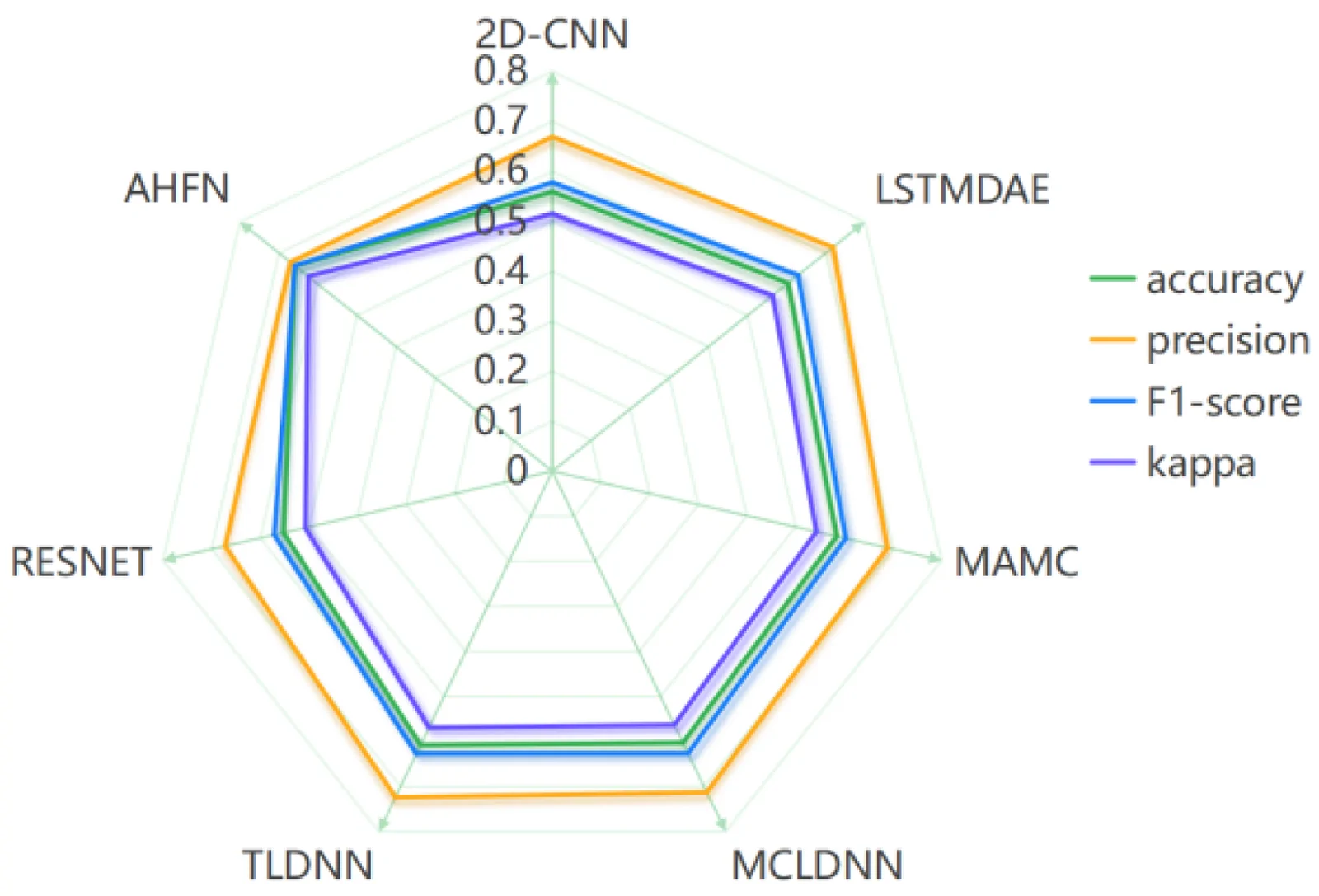
Model performance on RML2016.10A.

To address these limitations, this study proposes an Adaptive Holistic Fusion Network (AHFN) for robust low-SNR modulation recognition. The temporal graph is used as a fixed representation of local relationships between adjacent sampling points and is not regarded as the primary methodological contribution. Instead, AHFN progressively refines signal representations through three coordinated mechanisms. First, a Multi-Resolution Neural Fusion (MRNF) module employs parallel Kolmogorov–Arnold network branches with different spline-grid resolutions to extract complementary nonlinear waveform dynamics from standardized I/Q samples and temporal positional information. Second, an Adaptive Soft-Threshold Denoising (ASTD) module generates node- and channel-specific thresholds to suppress noise-sensitive responses while preserving weak modulation cues. Third, a Topology-Aware Multi-Scale Fusion Network (TAMSFN) performs feature- and structure-adaptive message aggregation over the fixed temporal graph and combines multi-scale graph representations with Mamba-based long-range sequence modeling. Therefore, the principal contribution of AHFN lies in multi-resolution nonlinear representation, adaptive noise suppression, and topology-aware local–global fusion rather than graph construction itself. A performance comparison of AHFN with representative baseline models on RML2016.10A is presented in Figure 1.

The remainder of this paper is organized as follows. Section 2 presents the system modeling and problem formulation. Section 3 introduces the materials and methods, including the overall architecture and principal modules of the proposed AHFN. Section 4 reports and discusses the experimental results, including comparative, ablation, parameter-sensitivity, limited-sample, and complexity analyses. Finally, Section 5 concludes the paper and outlines its limitations and future research directions.

## 2. System Modeling and Problem Formulation

In a wireless communication system, the received signal is affected by the modulation format, channel response, synchronization errors, and additive noise during transmission. The passband transmitted signal can be expressed as

(1) $$s\left(t\right)=A\left(t\right)\cos\;\left(2\pi f_{c}t+\phi \left(t\right)\right),$$

where A(t), ϕ(t), and $f_{c}$ denote the instantaneous amplitude, instantaneous phase, and carrier frequency, respectively. After propagation through the wireless channel, the received signal can be modeled as

(2) $$r\left(t\right)=\left(s(t)*h(t)\right)e^{j(2\pi \Delta ft+\theta )}+n\left(t\right),$$

where h(t) is the channel impulse response, Δf and θ denote the carrier frequency offset and phase offset, respectively, n(t) represents additive white Gaussian noise, and ∗ denotes convolution. At the receiver, the passband signal is down-converted into the complex baseband signal through quadrature demodulation:

(3) $$\begin{array}{cc} I(t) & =\mathrm{LPF}\;\left\{r\left(t\right)\cos\left(2\pi f_{c}t\right)\right\}, \end{array}$$

(4) $$\begin{array}{cc} Q(t) & =\mathrm{LPF}\;\left\{r\left(t\right)\sin\left(2\pi f_{c}t\right)\right\}, \end{array}$$

where LPF{·} denotes low-pass filtering. The corresponding complex baseband signal is given by

(5) x(t)=I(t)+jQ(t).

After analog-to-digital conversion, the continuous baseband signal is sampled as

(6) x[n]=I[n]+jQ[n],n=1,2,…,L,

where *L* is the number of samples within one observation window.

Based on the sampled I/Q sequence, the input of the proposed model is represented as

(7) $$X=\left[\begin{array}{cccc} I[1] & I[2] & \cdots & I[L]\\ Q[1] & Q[2] & \cdots & Q[L] \end{array}\right]\in R^{L\times 2}.$$

Given *X*, automatic modulation recognition (AMR) aims to identify the modulation type from a predefined label set $C=\{C_{1},C_{2},\ldots ,C_{K}\}$. Therefore, the recognition objective can be formulated as

(8) $$\hat{y}=\arg\underset{C_{k}\in C}{\text{max}}\;P\;\left(y=C_{k}|X\right),$$

where *y* and $\hat{y}$ denote the ground-truth and predicted modulation labels, respectively. Under low-SNR conditions, noise may weaken the temporal continuity, spectral characteristics, and I/Q trajectory structures of the sampled signal, resulting in fragmented representations and increasing the difficulty of robust modulation recognition.

## 3. Materials and Methods

**Figure 2:**
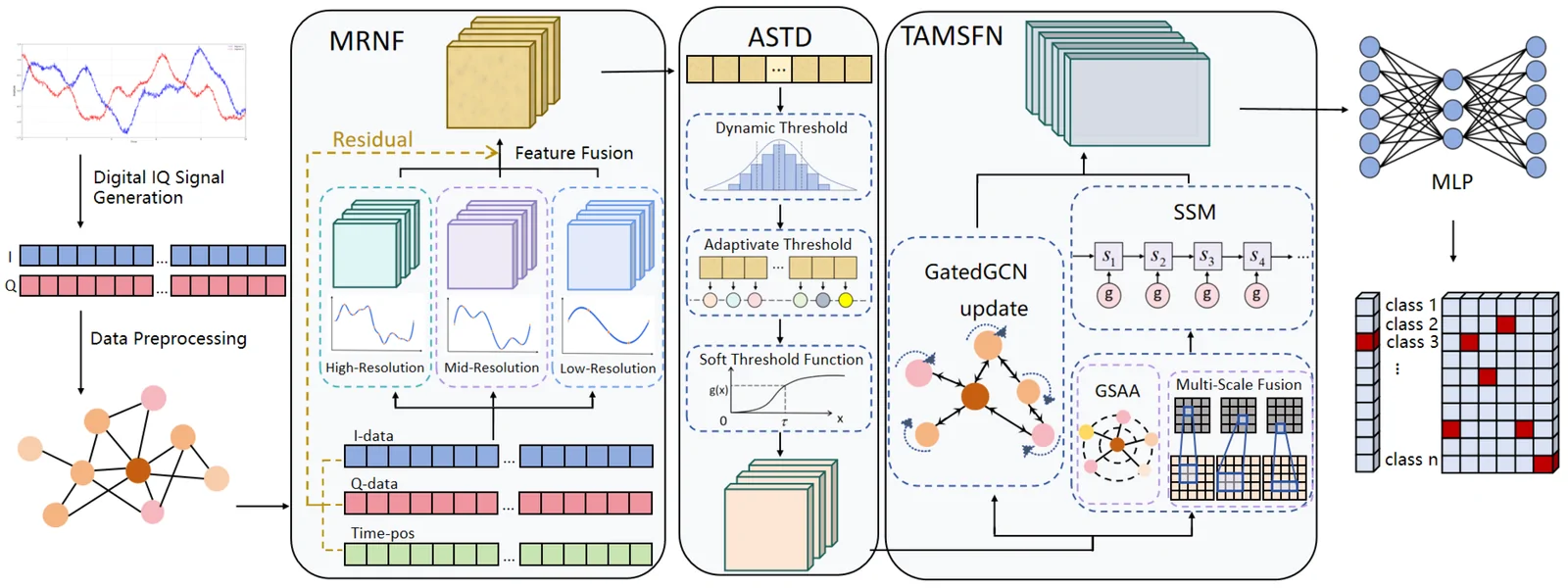
Overall architecture of the AHFN model.

To address the fragmentation of modulation-discriminative patterns under low-SNR conditions, this study proposes an Adaptive Holistic Fusion Network (AHFN) for robust automatic modulation recognition. As illustrated in Figure 2, each received I/Q segment is represented by a fixed temporal graph, where nodes correspond to temporal sampling points and edges describe predefined local temporal relationships. This graph representation provides a unified input structure rather than constituting the primary methodological contribution. Built upon the gated message-passing mechanism of GatedGCN [37], AHFN consists of a Signal Embedding Module, Multi-Resolution Neural Fusion (MRNF), Adaptive Soft-Threshold Denoising (ASTD), a Topology-Aware Multi-Scale Fusion Network (TAMSFN), and a Graph-Level Decision Module. Specifically, the Signal Embedding Module constructs the fixed temporal graph from standardized I/Q samples. MRNF then employs parallel KAN branches with different spline-grid resolutions to extract complementary nonlinear waveform dynamics from the I/Q observations and temporal positional information. ASTD generates node- and channel-specific thresholds to suppress noise-sensitive responses while preserving weak modulation cues. Subsequently, TAMSFN performs feature- and structure-adaptive message aggregation over the fixed graph and combines multi-scale graph representations with Mamba-based long-range sequence modeling. Finally, the Graph-Level Decision Module maps the refined representation to the corresponding modulation category.

### 3.1. Signal Embedding Module

The Signal Embedding Module is designed to transform the discrete I/Q sequence into a fixed temporal graph representation for subsequent structural feature learning. Different from directly treating the received signal as an ordered vector, the graph formulation explicitly describes local temporal neighborhoods and enables message passing among adjacent signal components. While CNN-, Transformer-, and Mamba-based models mainly focus on local feature extraction or sequential dependency modeling, graph representations provide a flexible mechanism for jointly capturing structural interactions and information propagation among neighboring signal samples. By representing signal samples as graph nodes and temporal adjacency as graph edges, the temporal evolution of the received signal is naturally encoded into the graph topology, allowing neighborhood relationships to be explicitly preserved and propagated during graph learning. This property is particularly beneficial under low-SNR conditions, where modulation-related characteristics are often fragmented by noise and weak local patterns can be reinforced through their temporal context rather than being learned independently. Given an input signal sample $X\in R^{L\times 2}$, where *L* denotes the sequence length and the two channels correspond to the in-phase and quadrature components, the signal is first standardized to reduce scale variation and improve training stability, where μ and σ denote the mean and standard deviation of the input features, respectively:

(9) $$Z=\frac{X-\mu }{\sigma },$$

Based on the standardized sequence, each time step is regarded as a graph node, and its feature is defined by the corresponding I/Q observation. To encode short-range temporal topology, bidirectional edges are constructed between each node and its *k* nearest temporal neighbors, with the edge attribute measured by the normalized temporal distance:

(10) $$\begin{array}{cccc} u_{t} & =\left[I_{t},Q_{t}\right]\in R^{2},\; & \; & t=1,2,\ldots ,L,\\ e_{t,t^{\prime }} & =\frac{|t-t^{\prime }|}{k}, & \; & t^{\prime }\in N_{k}\left(t\right). \end{array}$$

where $u_{t}$ denotes the node feature of time step *t*, $e_{t,t^{\prime }}$ denotes the edge attribute between nodes *t* and $t^{\prime }$, and $N_{k}\left(t\right)$ represents the *k*-nearest temporal neighborhood of node *t*. All node features form the node feature matrix $U\in R^{L\times 2}$, and the edge attributes form $E\in R^{|E|\times 1}$. Accordingly, the input signal is embedded as

(11) G=(V,E,U,E),

where V and E denote the node and edge sets, respectively. The adopted representation differs from several alternative signal structures. A chain graph can be regarded as a special case with k=1, in which each sampling point is connected only to its immediately adjacent samples, whereas the adopted temporal neighborhood provides a wider local receptive field while preserving temporal order. Unlike a learned graph, the proposed framework does not infer, add, or remove edges according to the input features. Compared with a conventional one-dimensional sequence model, the fixed temporal graph explicitly supports local neighborhood aggregation, while the subsequent Mamba pathway complements it by modeling long-range temporal dependencies. By encoding temporal adjacency into graph connections, the constructed graph preserves the local neighborhood relationships among consecutive I/Q samples and provides a stable structural prior for the subsequent feature-processing modules. The graph topology remains fixed after construction, whereas adaptive processing is introduced at the feature and message-propagation levels. Specifically, ASTD performs data-dependent soft-thresholding to suppress noise-sensitive node responses, while TAMSFN adaptively regulates information propagation over the fixed temporal connections according to node features, temporal edge attributes, structural relationships, and channel importance. This separation retains consistent temporal neighborhoods while allowing the model to refine node features and inter-node interactions according to the received signal.

### 3.2. Multi-Resolution Neural Fusion

After the discrete I/Q sequence is embedded into a topology-aware graph representation, the Multi-Resolution Neural Fusion (MRNF) module is introduced to enhance the continuity and discriminability of fragmented modulation features under low-SNR conditions. Modulation-dependent characteristics are jointly reflected in the instantaneous I/Q states and their evolution along the sampling axis. In particular, variations in waveform oscillation and phase progression provide important frequency-related cues for distinguishing different modulation formats. However, these patterns may occur over different temporal ranges and are easily obscured by noise. To address this issue, MRNF employs parallel KAN-based branches with different nonlinear representation granularities to capture complementary waveform dynamics. Fine-grained branches emphasize rapid local variations, such as abrupt phase transitions, pulse edges, and short-term oscillations, whereas coarse-grained branches characterize smoother patterns, including amplitude envelopes, long-range phase evolution, and slowly varying oscillatory trends. The resulting representations are subsequently integrated to provide a more comprehensive description of the received signal.

Given the standardized I/Q signal $X\in R^{L\times 2}$, MRNF first incorporates a normalized temporal encoding $T\in R^{L\times 1}$ to retain the sequential order of the received signal:

(12) $$X_{\mathrm{enh}}=\mathrm{concat}\left(X,T\right)\in R^{L\times 3},$$

By associating each I/Q sample with its temporal position, $X_{\mathrm{enh}}$ enables the subsequent nonlinear mappings to characterize not only the instantaneous signal state but also its evolution throughout the received segment. Since frequency-related modulation characteristics are manifested through waveform oscillations and phase changes across consecutive samples, the position-aware representation provides a suitable basis for learning local phase-transition patterns, oscillation-rate variations, and long-range temporal dependencies.

The enhanced representation is then processed by multiple KAN-based resolution branches:

(13) $$H_{\mathrm{KAN}}^{\left(i\right)}=f_{\mathrm{KAN}}^{\left(i\right)}\;\left(W^{\left(i\right)}\phi^{\left(i\right)}\left(X_{\mathrm{enh}}\right)\right),\;i=1,2,\ldots ,N,$$

where $W^{\left(i\right)}$ denotes the learnable projection matrix of the *i*-th branch, and $\phi^{\left(i\right)}\left(\cdot \right)$ denotes the nonlinear basis expansion implemented using the SiLU base function and B-spline functions. Since modulation-dependent waveform patterns may exhibit both smooth global evolution and rapid local variations, a single spline-grid resolution may be insufficient to characterize these complementary nonlinear structures. Therefore, MRNF employs three parallel KAN branches with grid sizes of 3, 5, and 7 over the fixed interval [−1,1], corresponding to coarse-to-fine spline-grid resolutions. All branches use a spline order of 2, a grid epsilon of 0.02, and scale settings of 0.05, 1, and 1 for the noise, base-function, and spline components, respectively. The spline grids remain fixed during training, while the network parameters and scalar gate of each branch are independently learned, with each gate initialized to 1. Except for the grid size, the remaining branch configurations are kept consistent. In this manner, the coarse-resolution branch favors smoother nonlinear trends, whereas the finer-resolution branches provide greater flexibility in characterizing localized waveform variations, allowing MRNF to learn complementary modulation representations across different nonlinear basis resolutions.

The outputs of the three KAN branches are concatenated to form a unified multi-resolution representation. Through the coarse-to-fine spline-grid resolutions, MRNF aggregates complementary nonlinear waveform information:

(14) $$H_{\mathrm{MR}}=\mathrm{concat}\left(H_{\mathrm{KAN}}^{\left(1\right)},H_{\mathrm{KAN}}^{\left(2\right)},\ldots ,H_{\mathrm{KAN}}^{\left(N\right)}\right),$$

The different grid sizes provide distinct nonlinear basis granularities, enabling the parallel branches to complement one another when characterizing smooth waveform trends and finer local variations in the position-enhanced I/Q sequence.

To prevent excessive nonlinear transformation from destroying the original I/Q prior, MRNF further introduces a residual projection pathway:

(15) $$H_{\mathrm{res}}=W_{p}X.$$

where $W_{p}$ is a learnable projection matrix. This residual pathway preserves the intrinsic structure of the original I/Q signal and provides a stable prior for feature fusion.

Finally, the multi-resolution nonlinear representation and the residual I/Q prior are adaptively fused:

(16) $$H_{\mathrm{out}}=\alpha H_{\mathrm{MR}}+\left(1-\alpha \right)H_{\mathrm{res}},\;\alpha \in \left[0,1\right],$$

where α is a learnable fusion coefficient that balances the multi-resolution nonlinear representation and the preserved I/Q prior. The three independently parameterized KAN branches employ different spline-grid resolutions, while their branch-specific scalar gates regulate their respective contributions. Consequently, MRNF integrates temporal positional information, complementary nonlinear waveform representations, and the original I/Q prior. This design strengthens the characterization of modulation-dependent waveform variations and provides a stable input for the subsequent adaptive denoising and topology-aware feature aggregation modules.

### 3.3. Adaptive Spatio-Temporal Denoising

To further improve the robustness of the fused representation, an Adaptive Spatio-Temporal Denoising (ASTD) module is introduced after MRNF. Although MRNF enhances modulation structures from multiple resolutions, the fused features may still contain noise-sensitive responses under low-SNR conditions. Conventional fixed-threshold shrinkage treats different input samples with the same denoising strength, which may suppress noise but also remove weak modulation cues that are critical for classification [38,39,40].

In contrast, ASTD introduces an input-adaptive denoising strategy, in which node- and channel-specific shrinkage thresholds are estimated from the fused representation itself. This enables the model to apply differentiated suppression to noise-sensitive responses while preserving weak but discriminative modulation structures.

Given the fused feature representation $H_{\mathrm{out}}$, ASTD first estimates an adaptive threshold through a lightweight gating mechanism:

(17) $$\tau =\sigma \left(W_{g}\left|H_{\mathrm{out}}\right|\right),$$

where σ(·) denotes the sigmoid activation and $W_{g}$ is a learnable linear projection. The threshold coefficient τ is generated from the magnitude of the current fused representation at the node and channel levels, allowing the suppression strength to vary with different feature responses rather than applying a shared threshold to all components.

Based on the adaptive threshold, ASTD performs feature-dependent soft-thresholding:

(18) $$H_{s}=\mathrm{sign}\left(H_{\mathrm{out}}\right)⊙\max\left(0,|H_{\mathrm{out}}|-\tau \beta \right),$$

(19) $$\tilde{H}=\sigma \left(W_{a}H_{s}\right)⊙H_{s},$$

where $H_{s}$ denotes the soft-thresholded representation, β is the threshold scaling factor set to 0.20, and $W_{a}$ denotes the learnable linear projection used to generate the feature-attention gate. The first operation applies differentiated shrinkage to individual node-channel responses, while the subsequent sigmoid gate further recalibrates the retained features according to their current representations. This coordinated processing reduces low-magnitude noise-sensitive responses without uniformly weakening all feature components.

To avoid over-denoising, ASTD further introduces an adaptive feature preservation mechanism:

(20) $$H=\alpha ⊙P\left(H_{\mathrm{out}}\right)+\left(1-\alpha \right)⊙\tilde{H},\;\alpha \in \left[0,1\right],$$

where P(·) denotes the residual projection, and α is a global learnable coefficient initialized to 0.7 and constrained to [0,1]. It balances the contributions of the residual representation and the denoised features during optimization. This design prevents weak but useful modulation cues from being excessively suppressed during denoising.

Through the above process, ASTD performs feature-adaptive refinement through node- and channel-specific threshold estimation, soft-threshold shrinkage, feature recalibration, and residual preservation. The adaptive operations are applied to the node representations rather than to the graph topology, enabling noise-sensitive responses to be attenuated while reducing the loss of weak modulation-related features. The refined representation is subsequently provided to TAMSFN for topology-aware message processing and local–global multi-scale fusion.

### 3.4. Topology-Aware Multi-Scale Fusion Network

After ASTD suppresses noise-sensitive responses, the denoised node features still require reliable structural interaction to recover modulation patterns fragmented under low-SNR conditions. Although the temporal graph provides stable local connections, directly aggregating neighboring features may propagate noise-induced pseudo-correlations when feature similarity becomes unreliable. To address this issue, the proposed Topology-Aware Multi-Scale Fusion Network (TAMSFN) introduces a feature- and structure-adaptive message-processing mechanism over the fixed temporal graph. Without altering the predefined graph topology, TAMSFN adaptively controls how information is selected, propagated, and emphasized through topology-guided attention, structure-aware message modulation, and channel-adaptive recalibration. The module jointly considers node-feature relevance, temporal edge priors, degree-related structural factors, inter-node feature differences, and channel importance, thereby strengthening topology-consistent modulation cues while reducing unreliable information propagation.

#### 3.4.1. Structure-Guided Graph Filtering

Specifically, TAMSFN first introduces a Graph Structure-Aware Attention (GSAA) mechanism to estimate the relevance of neighboring features over the fixed temporal graph. Unlike attention based solely on node-feature similarity, GSAA incorporates the temporal edge attribute into the attention score, allowing neighborhood aggregation to account for both feature relevance and the predefined temporal relationship. For a target node *i* and its neighbor *j*, the attention coefficient is formulated as

(21) $$\alpha_{ij}=\frac{\exp\left({\mathrm{LReLU}}_{0.2}\left(a^{T}\left[Ah_{i}\|Ah_{j}\right]+\lambda_{e}\phi \left(e_{ij}\right)\right)\right)}{\sum_{k\in N(i)}\exp\left({\mathrm{LReLU}}_{0.2}\left(a^{T}\left[Ah_{i}\|Ah_{k}\right]+\lambda_{e}\phi \left(e_{ik}\right)\right)\right)},$$

where $h_{i}$ and $h_{j}$ denote the denoised node features, N(i) denotes the fixed temporal neighborhood of node i, and A and a are learnable projection parameters. The scalar $\lambda_{e}$ controls the contribution of the projected temporal edge attribute $\phi (e_{ij})$; it is initialized to 1 and remains learnable during training. The attention scores are activated using LeakyReLU with a negative slope of 0.2 and normalized by softmax over the neighborhood of each target node. Thus, $\alpha_{ij}$ provides topology-guided attention without altering the predefined graph connections.

To further regulate the reliability of message propagation, a structure-aware modulation operator is dynamically generated from the temporal edge attribute, node-degree information, and inter-node feature difference:

(22) $$M_{ij}=\phi \left(E_{ij},d_{i},d_{j},\Delta h_{ij}\right),$$

where $E_{ij}$ denotes the projected temporal edge attribute, $d_{i}$ and $d_{j}$ denote the degrees of nodes *i* and *j*, respectively, and $\Delta h_{ij}=h_{i}-h_{j}$ represents their feature difference. In the implementation, the temporal edge attribute, logarithmically transformed node degrees, and inter-node feature difference are independently projected and combined with the incident node representations. A sigmoid function is subsequently applied to generate an edge-wise and channel-wise modulation operator $M_{ij}$. Thus, the graph connectivity remains fixed, while the message-passing strength over each existing edge is adaptively adjusted:

(23) $$h_{i}^{\prime }=Ah_{i}+\sum_{j\in N(i)}\alpha_{ij}\left(M_{ij}⊙Bh_{j}\right)$$

Here, $\alpha_{ij}$ evaluates neighboring-feature relevance under the temporal edge prior, while $M_{ij}$ performs structure-aware modulation of the corresponding message. The symbol ⊙ denotes element-wise multiplication between the message gate and the projected neighboring feature. The neighbor messages are aggregated by weighted summation and combined with the projected self-node feature $Ah_{i}$. Therefore, TAMSFN performs feature- and structure-adaptive message aggregation over the fixed temporal graph, emphasizing topology-consistent interactions while reducing the propagation of noise-induced responses.

In the implementation, the graph message-passing backbone consists of four gated graph convolution layers. Each layer incorporates batch normalization, ReLU activation, dropout with a rate of 0.4, and a residual connection.

After feature- and structure-adaptive message aggregation, TAMSFN further introduces channel-adaptive recalibration to emphasize modulation-sensitive feature dimensions and attenuate noise-prone responses. A graph-level channel descriptor is first obtained from the mean statistics of all nodes, and a lightweight gating network is then used to generate channel weights:

(24) $$\begin{array}{cc} z_{c} & =\frac{1}{N}\sum_{i=1}^{N}h_{i,c}^{\prime }, \end{array}$$

(25) $$\begin{array}{cc} g & =\sigma \left(W_{2}\delta \left(W_{1}z\right)\right), \end{array}$$

(26) $$\begin{array}{cc} h_{i}^{\prime \prime } & =g⊙h_{i}^{\prime }. \end{array}$$

where $h_{i,c}^{\prime }$ denotes the *c*-th channel of node *i*, $W_{1}$ and $W_{2}$ are learnable matrices, and δ(·) and σ(·) denote nonlinear activation and sigmoid functions, respectively. In the implementation, the graph-level channel descriptor is obtained by global mean pooling, and the channel-gating network consists of two linear layers with a reduction ratio of 4. The generated channel weights are broadcast to all nodes within the corresponding graph and applied through element-wise multiplication. The recalibrated features are subsequently processed by batch normalization, ReLU activation, dropout with a rate of 0.4, and a residual connection. This channel-adaptive recalibration emphasizes modulation-sensitive responses while attenuating noise-prone components. Through the coordinated use of topology-guided attention, structure-aware message modulation, and channel-adaptive recalibration, TAMSFN adaptively regulates feature selection, message propagation, and channel emphasis over the fixed temporal graph.

#### 3.4.2. Local–Global Multi-Scale Fusion

To further refine fragmented modulation representations, TAMSFN introduces a local–global multi-scale fusion strategy that couples scale-aware graph pooling with global sequence modeling. Under low-SNR conditions, discriminative cues may appear at different temporal granularities, ranging from short-term transients, such as phase jumps and waveform edges, to broader structural trends, such as amplitude envelopes and trajectory evolution. A single-scale graph representation may therefore overlook weak local details or fail to maintain global consistency. To address this, three parallel TopK pooling branches with pooling ratios of 0.4, 0.6, and 0.8 are adopted for scale-aware salient-node selection. The higher-ratio branch retains denser local details, whereas the lower-ratio branch provides a more compact structural representation. A normalized temporal coordinate is added to the pooled features, which are then restored to their original node positions according to the retained-node indices. The three aligned representations are concatenated to generate a fusion context through a linear projection followed by sigmoid activation. A shared linear scorer then produces scale scores, which are normalized by softmax across the scale dimension and used for weighted summation. The resulting multi-scale feature is denoted as $Z_{\mathrm{out}}$.

Although multi-scale pooling reorganizes salient modulation cues across resolutions, its modeling range is still constrained by graph neighborhoods and pooling hierarchies. To recover long-range temporal consistency, $Z_{\mathrm{out}}$ is further processed by a four-layer unidirectional Mamba stack:

(27) $$Z_{\mathrm{Mamba}}=\mathrm{Mamba}\left(Z_{\mathrm{out}}\right).$$

The Mamba pathway captures global dynamic correlations through state-space sequence modeling, complementing the locality of graph-based aggregation. In the implementation, the Mamba stack uses $d_{\mathrm{model}}=128$, $d_{\mathrm{state}}=16$, $d_{\mathrm{conv}}=4$, and expand=1, without bidirectional processing or explicit dropout inside the Mamba layers. Finally, the global sequence representation is fused with the local graph representation $Z_{\mathrm{GCN}}$ through learnable weighted fusion:

(28) $$Z_{\mathrm{final}}=\gamma Z_{\mathrm{gcn}}+\left(1-\gamma \right)Z_{\mathrm{Mamba}},$$

where γ is a global learnable coefficient initialized to 0.5 and constrained to [0,1] through a clamp operation. It provides a shared balance between the local graph representation and the global sequence representation and does not vary across input samples. The fused representation is subsequently processed by dropout with a rate of 0.4 and batch normalization. In this way, TAMSFN forms a scale-aware local–global refinement process: TopK pooling selects salient modulation cues at different retention ratios, cross-scale alignment preserves their temporal positions, scale-adaptive fusion integrates complementary representations, and Mamba captures long-range temporal dependencies.

The refined representation $Z_{\mathrm{final}}$ is finally delivered to the Graph-Level Decision Module, where node-level features are aggregated into a graph-level embedding and mapped to the modulation category space:

(29) $$\hat{y}=\arg\max_{C_{k}\in C}P\left(C_{k}|Z_{\mathrm{final}}\right).$$

Thus, the learned local structural dependencies and global temporal dynamics are jointly exploited for final modulation type recognition. The overall procedure of the proposed AHFN is summarized in Algorithm 1.
**Algorithm 1** Overall workflow of the proposed AHFN.**Require:** Raw I/Q signal samples *X***Ensure:** Predicted modulation label $\hat{y}$  1:G←SignalEmbedding(X)▹ Signal standardization and temporal graph construction  2:$H_{M}\leftarrow \mathrm{MRNF}\left(G\right)$▹ Multi-resolution nonlinear representation of position-aware I/Q features  3:$H_{D}\leftarrow \mathrm{ASTD}\left(H_{M}\right)$    ⁢                       ▹ Adaptive soft-threshold denoising**Topology-Aware Multi-Scale Fusion Network:**  4:$H_{G}\leftarrow \mathrm{TopologyAwareGraphFiltering}\left(H_{D},G\right)$        ▹ GatedGCN update with GSAA and structure-aware message modulation  5:$H_{P}\leftarrow \mathrm{MultiScaleTopKFusion}\left(H_{G},G\right)$      ▹ Multi-scale structural feature extraction  6:$H_{S}\leftarrow \mathrm{Mamba}\left(H_{P}\right)$             ▹ Long-range temporal dependency modeling  7:$H_{F}\leftarrow \mathrm{LocalGlobalFusion}\left(H_{P},H_{S}\right)$  8:$P\leftarrow \mathrm{GraphDecision}(H_{F})$  9:$\hat{y}\leftarrow \arg\max_{c}P_{c}$10:**return** $\hat{y}$

The principal parameter settings and feature dimensions used in the proposed AHFN are summarized in Table 1. The sequence length is determined by the input sample format, whereas the hidden dimension is kept consistent across the main network modules.

## 4. Results and Discussion

### 4.1. Datasets

This study evaluates the proposed AHFN on two widely used benchmark datasets for automatic modulation recognition, namely RML2016.10A and RML2016.10B, which are released by the DeepSIG platform [41,42]. Both datasets consist of synthetic wireless communication signals represented by I/Q samples under different modulation formats and signal-to-noise ratio (SNR) conditions. Each sample contains 128 complex baseband points and is represented as a two-channel I/Q sequence with the size of 2×128. RML2016.10A contains 11 modulation categories, including both analog and digital modulation types, with SNR values ranging from −20 dB to 18 dB. RML2016.10B provides a larger-scale benchmark with 10 modulation categories and the same SNR range, which is suitable for further evaluating model robustness and generalization under diverse noise levels. The detailed information of the two datasets is summarized in Table 2.

### 4.2. Experimental Setup

All experiments are conducted on the RML2016.10A and RML2016.10B datasets using a single NVIDIA RTX 4090 GPU. The input I/Q sequences are first standardized to zero mean and unit variance. The dataset is split into training (60%), validation (20%), and test (20%) sets. The AHFN model is trained for 120 epochs with a batch size of 128 using the AdamW optimizer. The initial learning rate is set to 0.001 and decayed automatically when the validation performance stagnates, with a lower bound of $1\times 10^{-6}$. To evaluate the effectiveness of the proposed AHFN framework, comparisons are made against several representative state-of-the-art models, covering convolutional, recurrent, and hybrid architectures: 2D-CNN [41], ResNet [43], LSTMDAE [44], MCLDNN [45], TLDNN [46], and MAMC [47]. These baselines provide comprehensive benchmarks for assessing the capability of feature extraction, temporal modeling, and multi-domain fusion under varying SNR conditions.

### 4.3. Experimental Analysis

#### 4.3.1. Overall Performance and Complexity Analysis

**Figure 3:**
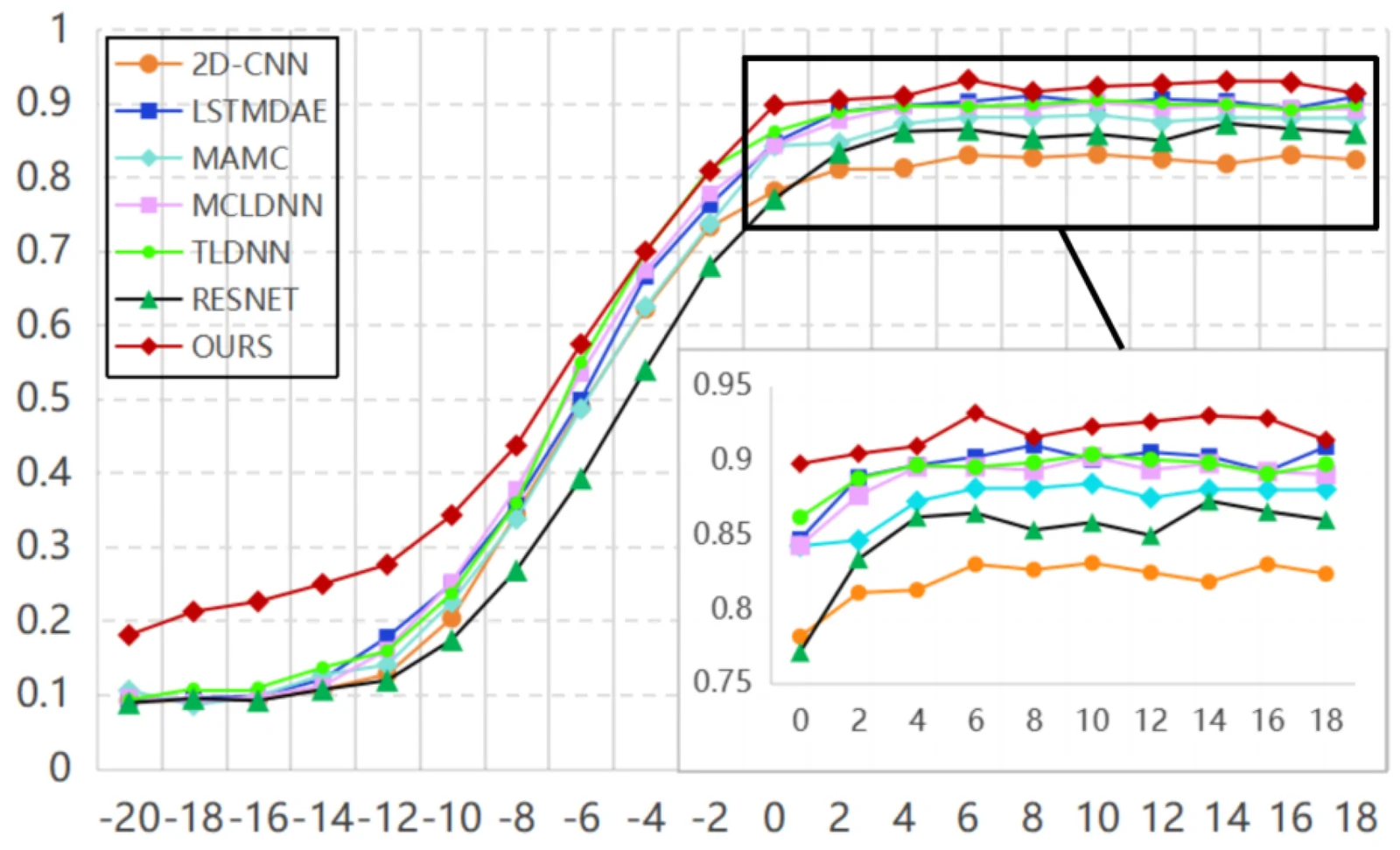
Accuracy vs. SNR for AHFN and baseline models on RML2016.10A.

On the RML2016.10A dataset (Table 3 and Figure 3), AHFN achieves an overall accuracy of 65.93% and shows favorable performance relative to the compared methods. The advantages are more noticeable in the low-SNR range (−20 dB to 0 dB), where the received signals suffer from severe feature degradation and fragmentation. These observations suggest that the proposed architecture is effective in extracting discriminative modulation characteristics under challenging noise conditions. The multi-resolution neural fusion promotes complementary interactions among I/Q, temporal, and spectral information, whereas the adaptive soft-threshold denoising module helps reduce noise-sensitive responses while retaining informative signal structures. Furthermore, the topology-aware local–global fusion captures both local feature dependencies and long-range temporal relationships. Together, these components contribute to more coherent feature representations and improved modulation recognition performance, particularly in low-SNR scenarios. It should be noted that Figure 3 reports the classification accuracy at each individual SNR, whereas Table 3 presents the average performance over all evaluated SNR levels. Therefore, the accuracy at some individual SNR levels in the figure may be higher than the overall average reported in the table.

**Figure 4:**
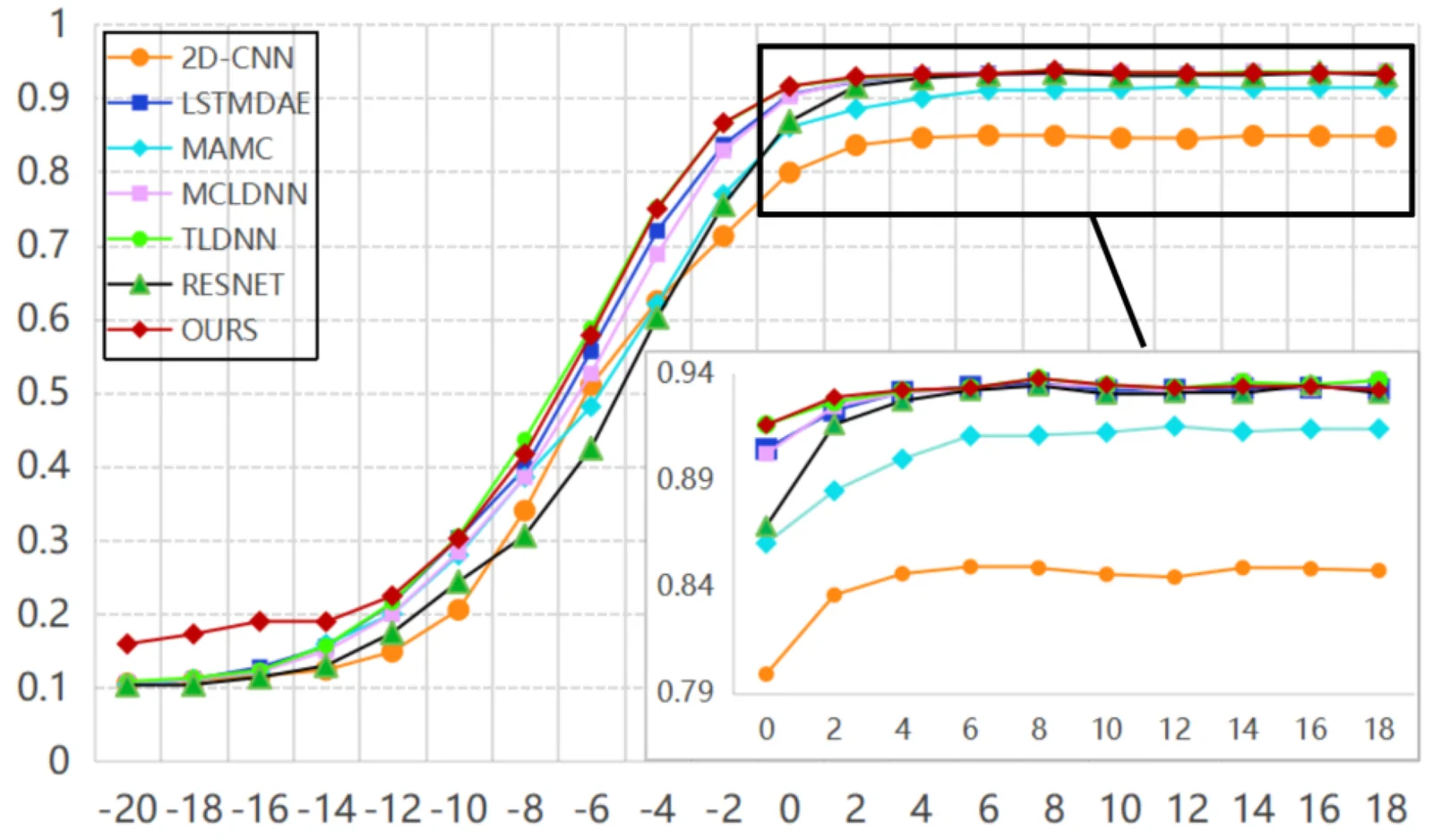
Accuracy vs. SNR for AHFN and baseline models on RML2016.10B.

On the RML2016.10B dataset (Table 4 and Figure 4), AHFN achieves an overall accuracy of 65.82% and demonstrates favorable performance relative to the compared methods. The performance improvements are more noticeable in the low-SNR region, where modulation-related features are severely degraded and fragmented by noise. These observations suggest that the proposed architecture is effective in preserving and integrating informative signal characteristics under challenging conditions. Specifically, the multi-scale pooling strategy facilitates the aggregation of salient nodes across different resolutions, while the Mamba-based sequence modeling captures long-range temporal dependencies. In addition, the structure-guided graph filtering promotes topology-aware feature propagation and aggregation. Together, these components contribute to more coherent graph-level representations, which are associated with improved recognition performance and more reliable predictions across different modulation categories. It should be noted that Figure 4 reports the classification accuracy at each individual SNR, whereas Table 4 summarizes the average performance over all evaluated SNR levels. Therefore, the accuracy at some individual SNR levels in the figure may be higher than the overall average reported in the table.

As shown in Table 5, AHFN has the largest parameter count and FLOPs among the evaluated models, mainly due to its multi-resolution nonlinear representation, adaptive denoising, and topology-aware multi-stage feature fusion. However, its average GPU latency is 25.23 ms, which is lower than those of MCLDNN, ResNet, and LSTM-DAE, although higher than those of 2D-CNN, TLDNN, and MAMC. This indicates that theoretical computational complexity does not increase inference latency proportionally, likely because GPU parallelism and operator implementation efficiency also affect runtime performance. In terms of memory consumption, AHFN requires 35.91 MB, which is lower than TLDNN but higher than the other baselines. Overall, AHFN trades model compactness and computational cost for stronger feature representation and recognition performance, particularly under low-SNR conditions, while further lightweight optimization through module simplification, parameter sharing, pruning, or knowledge distillation remains a relevant direction for future work.

#### 4.3.2. Model Stability and Monte Carlo Analysis

**Figure 5:**
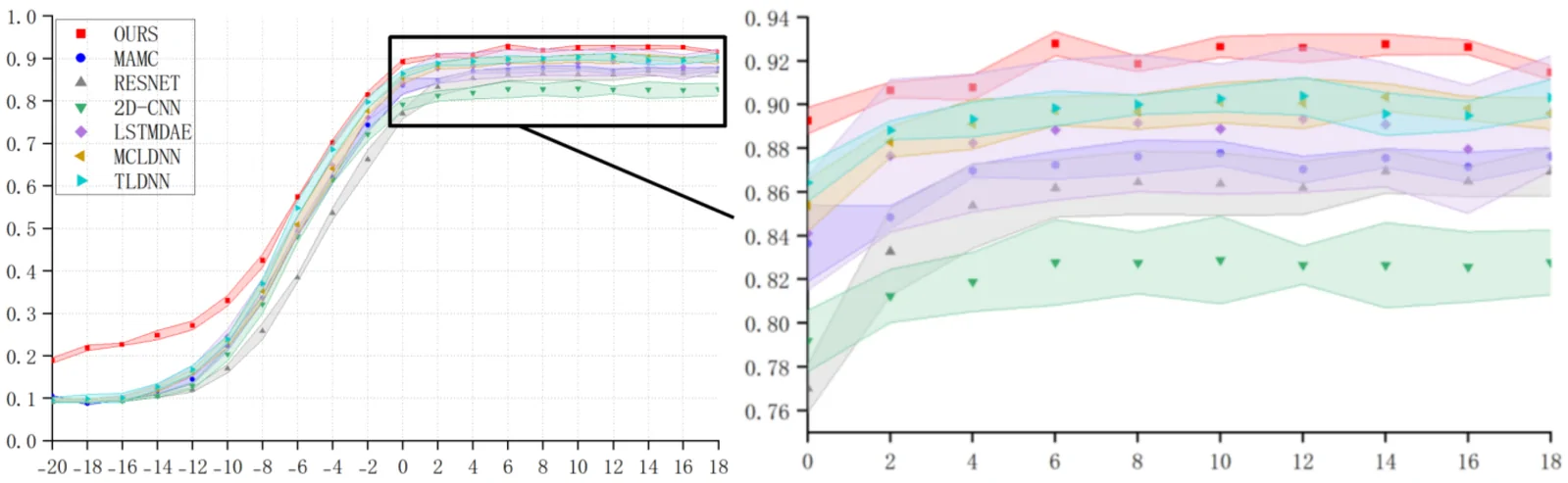
Monte Carlo evaluation of AHFN and baseline models on RML2016.10A.

**Figure 6:**
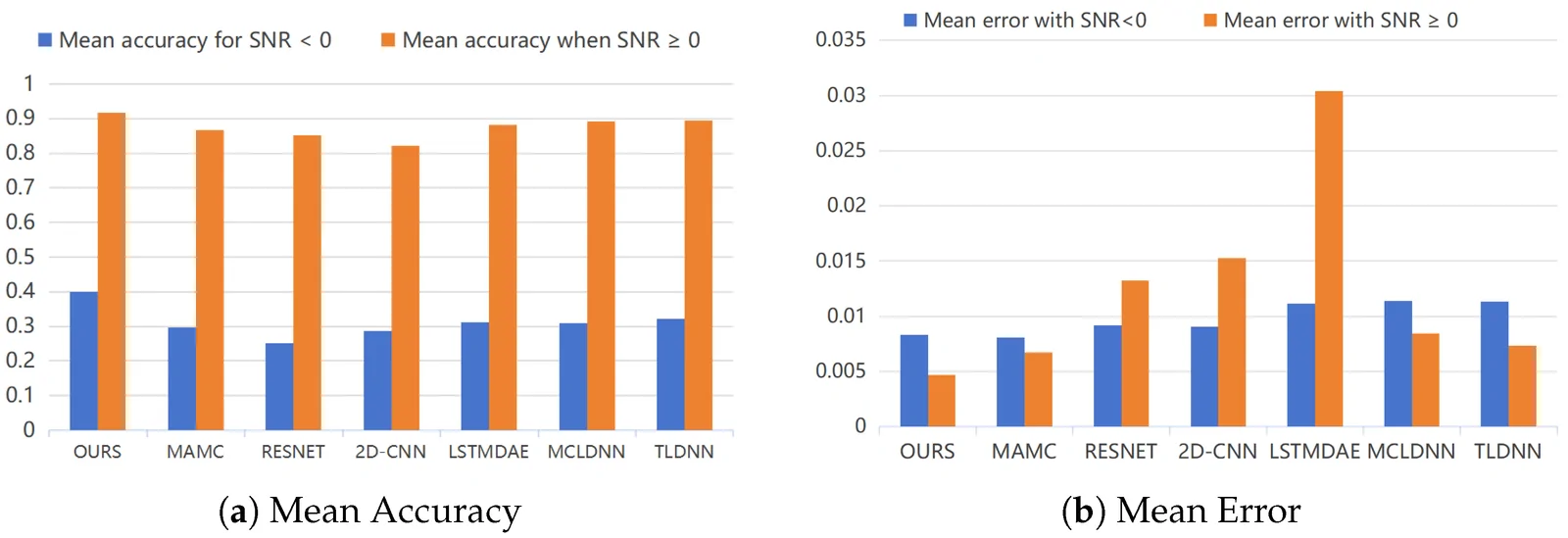
Statistical evaluation of accuracy and error for Monte Carlo experiments on RML2016.10A.

The Monte Carlo evaluation in Figure 5 and the statistical results in Figure 6 demonstrate that AHFN consistently outperforms all baseline models across SNR levels, with the most significant gains observed in low-SNR conditions (−20 dB to 0 dB). Specifically, the mean accuracy of AHFN under negative SNR values is markedly higher than other methods, indicating its superior ability to preserve discriminative features when signals are heavily corrupted by noise. Concurrently, the mean error remains the lowest across trials, suggesting enhanced stability and robustness in repeated stochastic evaluations. This performance can be attributed to AHFN’s key architectural innovations: the multi-resolution neural fusion module effectively aligns I/Q, temporal, and spectral features, mitigating fragmentation and improving cross-domain feature continuity; the adaptive soft-threshold denoising module selectively suppresses noise while retaining weak modulation cues, preventing over-smoothing of subtle signal characteristics; and the topology-aware local–global fusion ensures that both short-range structural dependencies and long-range temporal correlations are captured, providing coherent and discriminative graph-level representations. Collectively, these mechanisms enable AHFN to maintain high accuracy and low variance under challenging noise conditions, demonstrating both robustness and reliability beyond conventional convolutional, recurrent, and hybrid architectures.

#### 4.3.3. Class-Level Recognition Analysis

**Figure 7:**
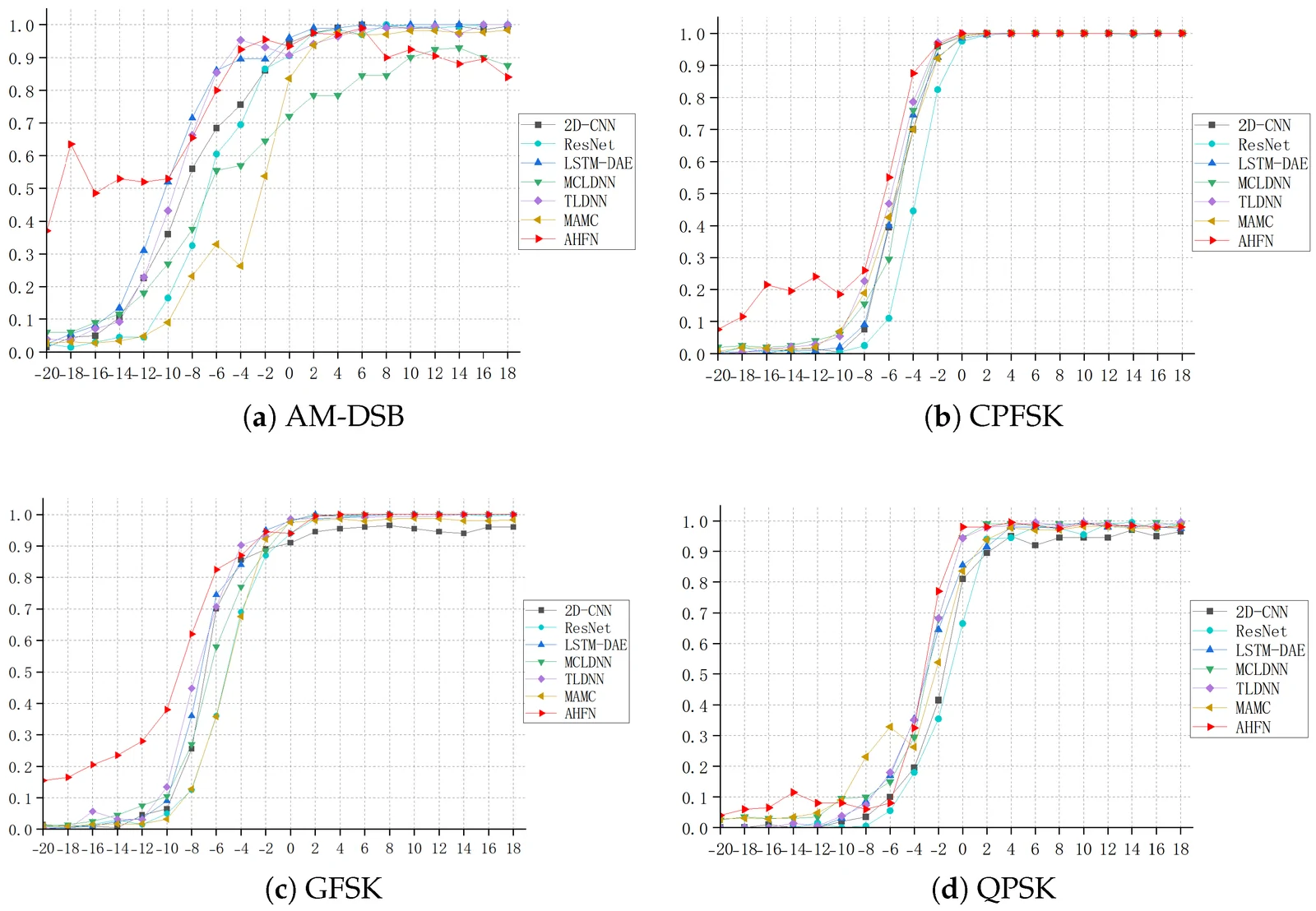
Class-wise accuracy comparison of different models on selected modulation types.

As shown in Figure 7, the proposed AHFN exhibits clear advantages on the selected modulation types, especially in the low- and medium-SNR regions where discriminative structures are easily distorted by noise. For AM-DSB, AHFN maintains a relatively high recognition accuracy even at extremely low SNRs, indicating that the proposed adaptive denoising and multi-resolution fusion can better preserve amplitude-related modulation cues. For CPFSK and GFSK, AHFN shows faster accuracy recovery as SNR increases from approximately −10 dB to 0 dB, suggesting stronger capability in capturing continuous frequency- and phase-related temporal structures. For QPSK, AHFN achieves more stable performance in the transition region from low to moderate SNR, where phase ambiguity usually leads to severe class confusion. These results further demonstrate that AHFN is not only effective in overall accuracy improvement but also provides stronger class-level robustness for modulation types with different signal characteristics.

**Figure 8:**
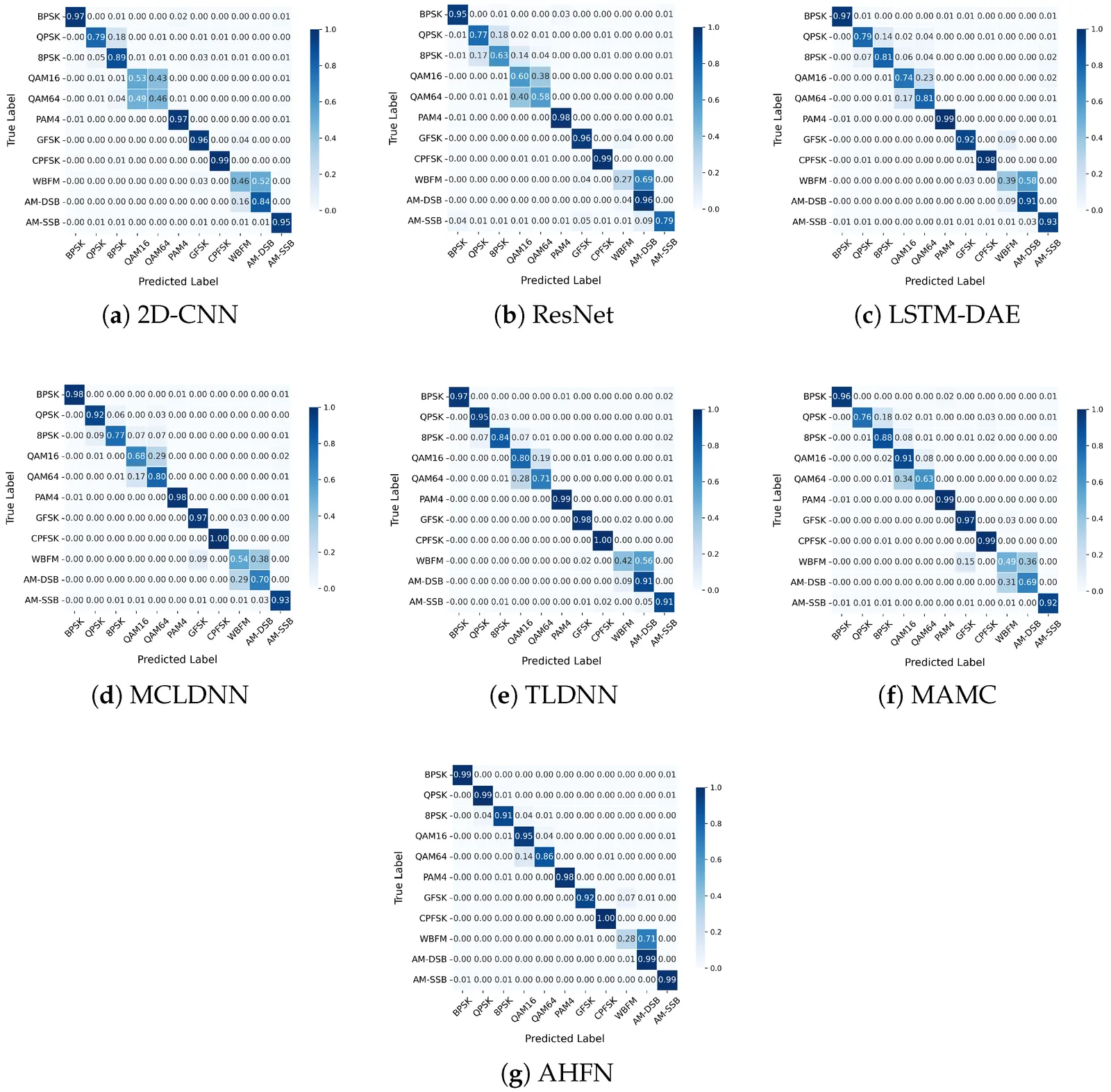
Confusion matrices of different models on RML2016.10A at 0 dB.

As shown in Figure 8, the confusion matrices at 0 dB further verify the class-level discriminability of the proposed AHFN. Compared with baseline models, AHFN presents a more compact diagonal distribution, indicating that the predicted labels are more consistent with the true modulation categories. For modulation types with relatively stable structures, such as BPSK, QPSK, PAM4, GFSK, and CPFSK, most methods achieve high recognition accuracy, whereas AHFN maintains reliable classification with fewer off-diagonal errors. More importantly, for challenging categories such as QAM16 and QAM64, where dense constellation distributions easily lead to inter-class confusion, AHFN shows clearer separation than conventional CNN-, recurrent-, and hybrid-based models. In addition, the confusion between AM-DSB, AM-SSB, and WBFM is alleviated to some extent, suggesting that the proposed multi-resolution fusion, adaptive denoising, and topology-aware local–global modeling can better preserve weak amplitude- and frequency-related cues under noisy conditions. The corresponding confusion matrices at −4 dB and −2 dB are provided in Appendix A for a more complete comparison under lower-SNR conditions. Overall, the results demonstrate that AHFN improves not only overall recognition accuracy but also fine-grained class separability, especially for modulation types with overlapping or noise-sensitive feature distributions.

#### 4.3.4. Ablation Study of the AHFN Model

- Component Ablation

**Figure 9:**
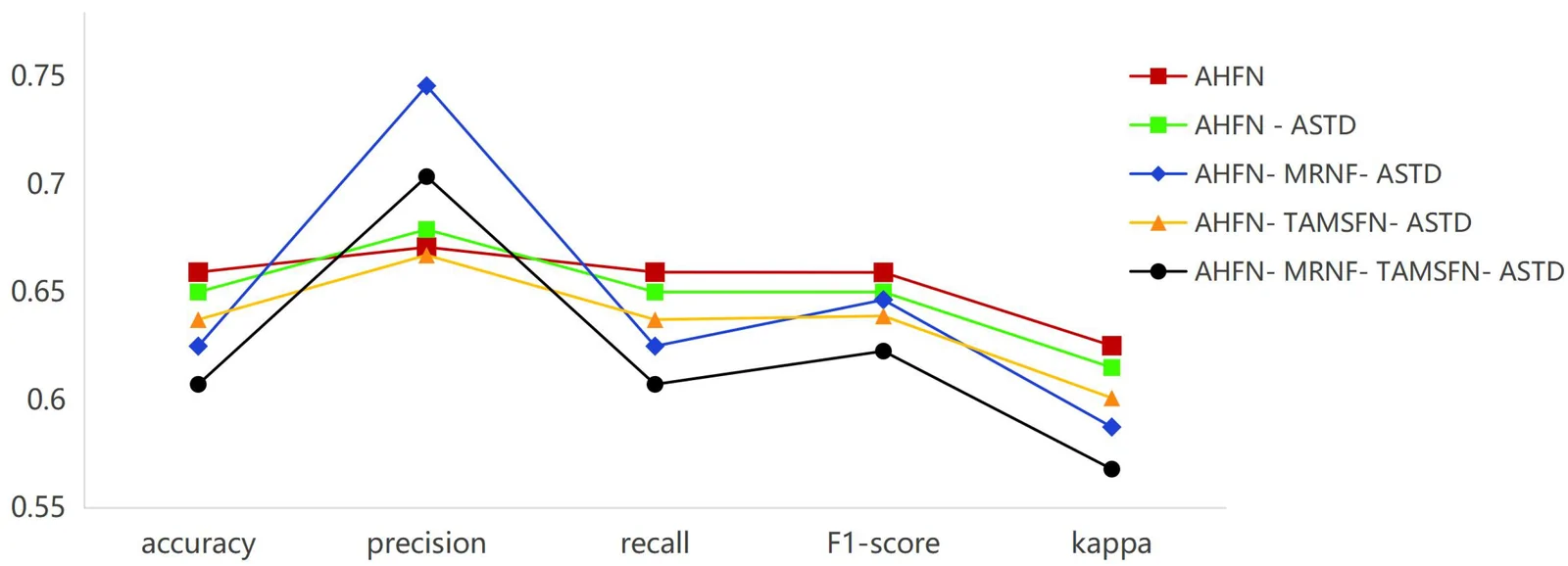
Line chart of ablation study metrics on the RML2016.10A dataset.

To quantify the contribution of each core module in AHFN, ablation experiments were conducted on the RML2016.10A dataset, with the results summarized in Figure 9 and Table 6. Removing the Adaptive Spatio-Temporal Denoising (ASTD) module reduced the accuracy by 0.92%, demonstrating its effectiveness in suppressing noise while preserving weak modulation-related cues. Excluding the *Multi-Resolution Neural Fusion* (MRNF) module resulted in a further 2.51% decrease, confirming its importance in capturing and integrating multi-resolution nonlinear waveform dynamics under low-SNR conditions. Omitting the *Topology-Aware Multi-Scale Fusion Network* (TAMSFN) led to a 1.77% accuracy reduction, indicating its contribution to modeling structural correlations and achieving coherent multi-scale feature aggregation. Overall, the complete AHFN improves accuracy by 5.2% over the baseline, showing that adaptive denoising, multi-resolution nonlinear feature fusion, and topology-aware aggregation jointly contribute to robust modulation recognition under challenging low-SNR conditions.

b. Design Choice Comparison

To further examine the rationale behind the principal architectural choices, we compare alternative graph constructions and component implementations while keeping the remaining network structure and training settings unchanged. Specifically, the adopted temporal graph is compared with a chain graph and a feature-based KNN graph, while KAN, adaptive thresholding, structure-aware graph processing, and Mamba are respectively replaced by an MLP, fixed thresholding, standard GCN, and GRU. The corresponding results are presented in Table 7.

As shown in Table 7, replacing the principal designs of AHFN consistently reduces the overall recognition performance compared with the complete model reported in the preceding module-level ablation study. The lower results of A-CHAIN and A-KNN indicate that the adopted fixed temporal graph provides a more suitable neighborhood structure for preserving local temporal continuity than either immediate-neighbor connections or feature-based KNN construction. A-MLP obtains relatively high precision but lower accuracy, recall, F1-score, and Kappa, suggesting that the KAN-based encoder provides a more balanced representation of weak modulation patterns. The performance degradation of A-FIX further supports the use of node–channel adaptive thresholding for data-dependent suppression of noise-sensitive responses. Similarly, the results of A-GCN indicate that the proposed structure-aware graph processing is more effective than conventional GCN-based aggregation in regulating message propagation over the fixed temporal graph. Although A-GRU achieves the strongest overall performance among the replacement variants, it remains inferior to the complete AHFN, supporting the contribution of Mamba-based sequence modeling to capturing long-range temporal dependencies. Overall, these comparisons demonstrate that the selected graph construction, nonlinear representation, adaptive denoising, structure-aware message processing, and global sequence modeling jointly contribute to the robustness of AHFN.

#### 4.3.5. Hyperparameter Sensitivity Analysis

- Impact of KAN Branch Quantity on Multi-Resolution Feature Extraction

**Figure 10:**
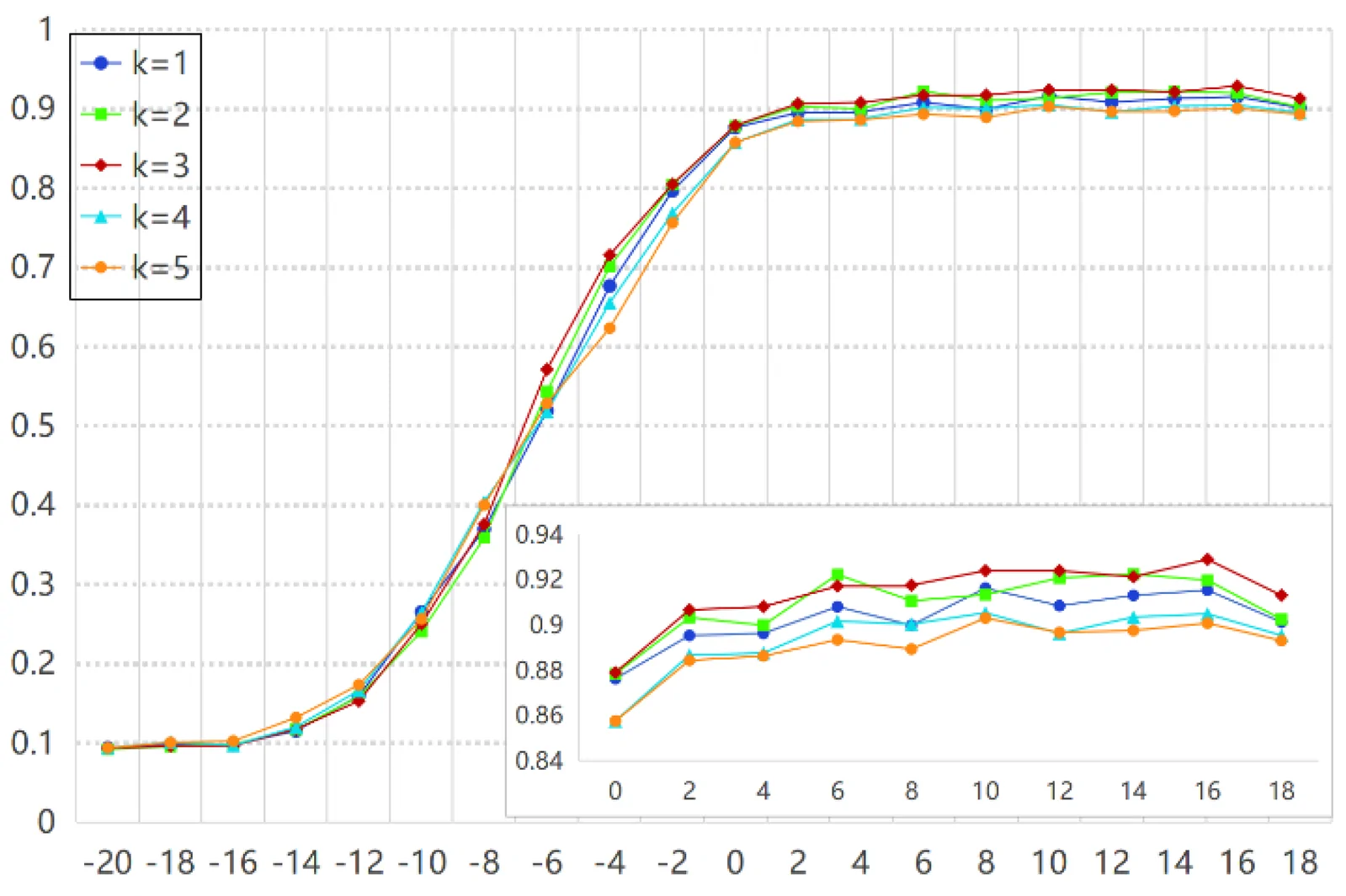
Effect of varying the number of KAN branches on modulation recognition performance.

To investigate the influence of branch count in the multi-resolution KAN architecture, models with different numbers of parallel branches (k=1–5) were evaluated on modulation recognition tasks. As shown in Figure 10, increasing the number of branches initially improves feature representation and classification accuracy by capturing richer multi-scale signal structures. However, configurations with excessive branches (k=4 or 5) exhibit slight performance degradation, likely due to overfitting and challenges in joint optimization. Detailed analysis across SNR levels reveals that higher branch counts offer marginal gains under very low SNR conditions (≤−8 dB), while a moderate configuration (k=3) achieves the best balance between expressive capacity and generalization at higher SNRs. Single- or dual-branch setups fail to capture sufficient multi-scale details, limiting discriminative power. These results validate the three-branch design as optimal for the MRNF module, ensuring stable training, effective multi-resolution feature extraction, and robust performance under varying noise conditions.

b. Sensitivity Analysis of the Three-Level TopK Ratio

As shown in Table 8, the accuracy, recall, F1-score, and Kappa coefficient initially increase and then decrease as the three-level TopK pooling ratios increase. The setting [0.4,0.6,0.8] achieves the best overall performance, with an accuracy of 65.93%, an F1-score of 65.92%, and a Kappa coefficient of 62.52%. Although larger retention ratios yield higher precision, they do not lead to corresponding improvements in the other metrics, suggesting that retaining more nodes does not necessarily improve the overall discriminative ability of the model. In contrast, excessively small ratios may result in the loss of useful structural information. Therefore, [0.4,0.6,0.8] provides a more suitable balance between feature preservation and graph compression and is adopted as the default configuration in TAMSFN.

#### 4.3.6. Few-Shot Recognition Performance

To further evaluate the data efficiency and robustness of the proposed method under limited labeled data, few-shot experiments were conducted using four training-sample settings, namely 10, 50, 100, and 200 samples for each modulation class–SNR combination. AHFN, MCLDNN, and TLDNN were trained and evaluated under identical experimental settings.

As shown in Table 9, although the performance of all models improves as the number of training samples increases, the proposed AHFN exhibits a more pronounced advantage when labeled data are severely limited. Under the 10-shot setting, AHFN surpasses the strongest baseline by 12.27, 19.44, 13.50, and 5.20 percentage points in accuracy, macro-precision, Kappa, and low-SNR accuracy, respectively, whereas these margins decrease to 3.09, 5.71, 3.39, and 0.98 percentage points under the 200-shot setting. This narrowing performance gap indicates that the comparison models can partially compensate for their representation limitations when more labeled samples are available, while AHFN is more effective at extracting discriminative information from scarce training data. In particular, the larger improvement in macro-precision than in overall accuracy suggests that AHFN more effectively suppresses class-wise false-positive predictions under extreme sample scarcity, rather than improving only a few dominant modulation categories. Meanwhile, the consistent trend between accuracy and Kappa confirms that the observed gains are not caused merely by chance agreement or an imbalanced prediction distribution. Although the advantage in low-SNR accuracy gradually decreases with increasing sample size, AHFN remains superior in all settings, demonstrating that its feature extraction and denoising mechanisms are especially beneficial when limited labeled data and severe noise degradation occur simultaneously. Furthermore, the relatively small error bars under the most challenging 10-shot setting indicate that AHFN produces more consistent results across repeated independent runs, whereas the comparison models show considerably larger fluctuations. Overall, these results demonstrate that AHFN provides stronger sample efficiency, more reliable class-level recognition, and better performance consistency under few-shot conditions.

#### 4.3.7. Visualization of Learned Feature Representations

To further investigate how different modules progressively improve feature representation, t-SNE is employed to visualize the intermediate features extracted from the MRNF, ASTD, and TAMSFN modules, as well as the final fused representation. The high-dimensional features are projected into a two-dimensional space, where samples from different modulation categories are distinguished by different colors. This visualization enables a qualitative comparison of the class distribution, intra-class compactness, and inter-class separability at different stages of the proposed network, thereby illustrating the contribution of each module to discriminative feature learning.

**Figure 11:**
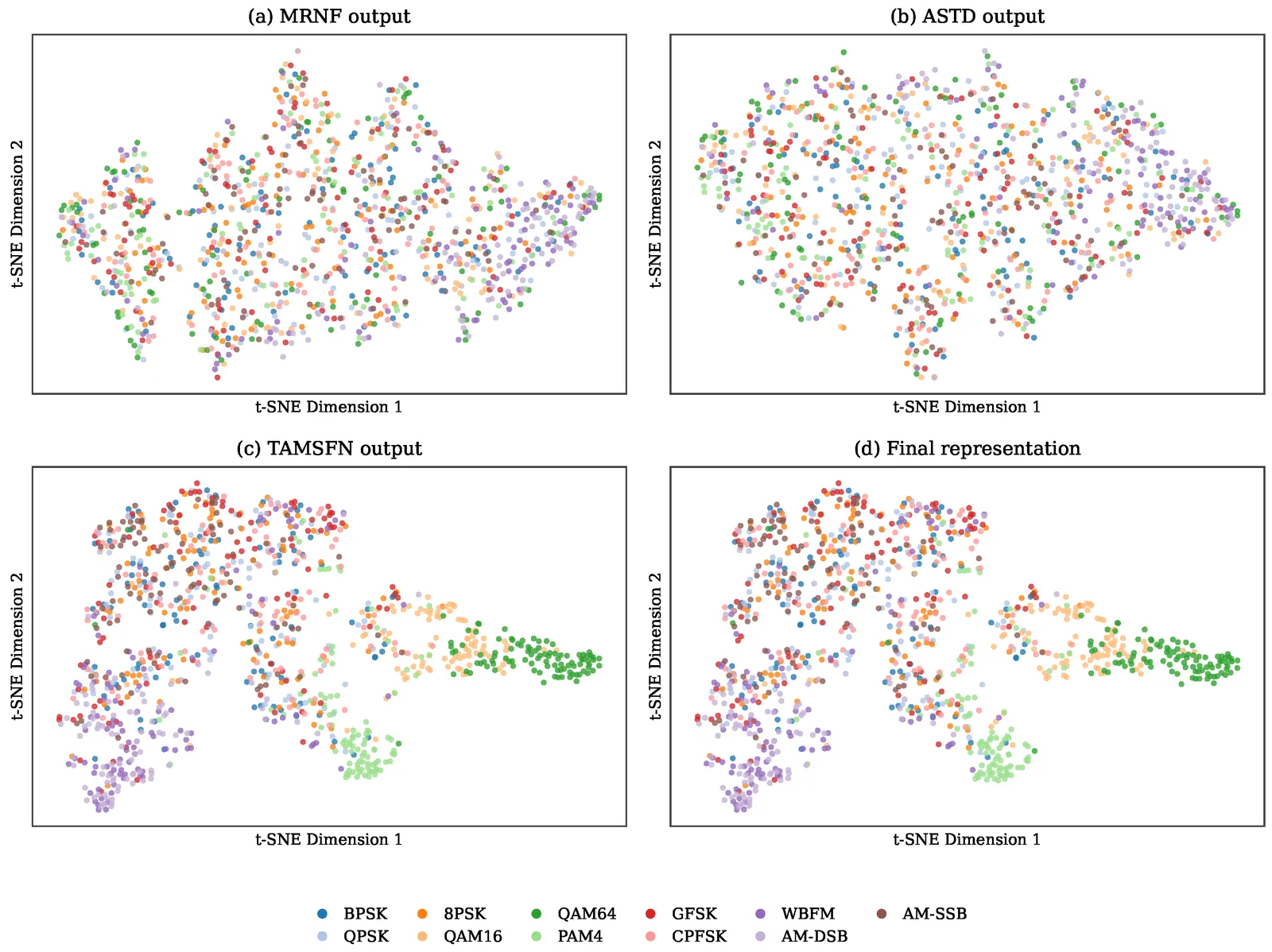
t-SNE visualization of the feature representations generated at different stages of the proposed network: (**a**) MRNF output, (**b**) ASTD output, (**c**) TAMSFN output, and (**d**) final representation. Different colors denote different modulation categories.

As shown in Figure 11, the features produced by MRNF and ASTD remain largely intermixed, indicating that the early modules mainly enhance and denoise the input representations but do not yet establish clear class boundaries. After TAMSFN processing, several modulation categories form more compact and separable clusters, demonstrating its effectiveness in modeling discriminative temporal and structural information. The final representation further improves the intra-class compactness and inter-class separation, particularly for QAM64, PAM4, and WBFM, although partial overlap remains among several similar modulation types. Overall, the progressive evolution of the feature distributions confirms that the proposed modules collaboratively transform the initially mixed features into more discriminative representations.

## 5. Conclusions

This study proposes an Adaptive Holistic Fusion Network (AHFN) for robust automatic modulation recognition under low-SNR conditions. AHFN combines multi-resolution nonlinear representation, adaptive soft-threshold denoising, and topology-aware local–global fusion over a fixed temporal graph. Specifically, MRNF extracts complementary waveform dynamics through parallel KAN branches, ASTD suppresses noise-sensitive responses using node- and channel-specific thresholds, and TAMSFN integrates local structural interactions with long-range temporal dependencies. Experimental results on RML2016.10A and RML2016.10B demonstrate that AHFN achieves favorable recognition performance under severe noise, while the ablation and stability analyses further support the effectiveness of its principal components. The current evaluation is limited to benchmark datasets with fixed signal lengths and predefined modulation categories and does not explicitly consider cross-device mismatch, open-set signals, or deliberately optimized adversarial perturbations. Future work will investigate lightweight model design, cross-device generalization, open-set recognition, and adversarial robustness to further improve the practical applicability of AHFN.

## Figures and Tables

**Table 1:** Key parameter settings and feature dimensions of the proposed AHFN.

Component	Parameter Setting	Feature Dimension
Input signal	Sequence length *L*	$X\in R^{L\times 2}$
Temporal encoding	One normalized position channel	$T\in R^{L\times 1}$, $X_{\mathrm{enh}}\in R^{L\times 3}$
Temporal graph	N=L, neighborhood range k=7	*N* nodes with temporally connected edges
MRNF	Three KAN branches with resolutions {3,5,7}; spline order 2	Each branch: $H_{\mathrm{KAN}}^{\left(i\right)}\in R^{N\times d}$
Unified hidden representation	Hidden dimension d=128	$H\in R^{N\times 128}$
ASTD	Threshold scaling factor 0.20; initial residual coefficient $\alpha_{0}=0.7$	$H_{D}\in R^{N\times 128}$
TAMSFN	Four stacked layers; multi-scale pooling ratios {0.4,0.6,0.8}	Output of each layer: $R^{N\times 128}$
Mamba	$d_{\mathrm{state}}=16$, $d_{\mathrm{conv}}=4$, expansion factor 1	$H_{S}\in R^{N\times 128}$
Local–global fusion	Initial fusion coefficient $\gamma_{0}=0.5$	$H_{F}\in R^{N\times 128}$
Graph decision	Mean pooling followed by an MLP	$P\in R^{C}$, C=11/10 for RML2016.10A/10B

**Table 2:** Details of the RML2016.10A and RML2016.10B datasets.

Dataset	RML2016.10A	RML2016.10B
Source	DeepSIG	DeepSIG
Signal format	I/Q samples	I/Q samples
Input size	2×128	2×128
Number of modulation classes	11	10
Modulation types	BPSK, QPSK, 8PSK,	BPSK, QPSK, 8PSK,
	QAM16, QAM64, CPFSK,	QAM16, QAM64, CPFSK,
	GFSK, PAM4, AM-DSB, AM-SSB, WBFM	GFSK, PAM4, AM-DSB, WBFM
SNR range	−20 dB to 18 dB	−20 dB to 18 dB
SNR interval	2 dB	2 dB
Number of samples	220,000	1,200,000
Task	Modulation classification	Modulation classification

**Table 3:** Performance comparison on RML2016.10A.

Model	Accuracy [48]	Precision [48,49]	Recall [48,49]	F1-Score [48]	Kappa
2D-CNN	0.5590	0.6690	0.5590	0.5779	0.5149
ResNet	0.5523	0.6722	0.5523	0.5712	0.5075
LSTMDAE	0.6038	0.7189	0.6038	0.6292	0.5642
MCLDNN	0.6037	0.6649	0.6037	0.6193	0.5640
TLDNN	0.6082	0.7238	0.6082	0.6275	0.5690
MAMC	0.5843	0.6885	0.5842	0.6029	0.5427
AHFN	0.6593	0.6709	0.6593	0.6592	0.6252

**Table 4:** Performance comparison on RML2016.10B.

Model	Accuracy	Precision	Recall	F1-Score	Kappa
2D-CNN	0.5706	0.6590	0.5706	0.5747	0.5228
ResNet	0.6096	0.6575	0.6096	0.6148	0.5662
LSTMDAE	0.6409	0.6893	0.6409	0.6430	0.6010
MCLDNN	0.6349	0.6668	0.6349	0.6395	0.5943
TLDNN	0.6492	0.6856	0.6492	0.6519	0.6102
MAMC	0.6133	0.6449	0.6133	0.6156	0.5703
AHFN	0.6582	0.6867	0.6582	0.6609	0.6202

**Table 5:** Computational complexity comparison of different models.

Model	Parameters (M)	FLOPs (G)	Avg. GPU Latency (ms)	Peak GPU Memory (MB)
2D-CNN	2.830427	0.038329	8.041959	10.933838
ResNet	0.08552	0.003240	90.131136	0.337646
LSTM-DAE	0.014829	0.000021	97.210542	16.189697
MCLDNN	0.405175	0.035774	28.352904	10.729492
TLDNN	0.243340	0.007890	1.304555	41.774414
MAMC	0.535243	0.003137	1.835064	11.222656
AHFN	3.118535	0.795406	25.231048	35.905762

**Table 6:** Ablation study of different modules.

Methods	MRNF	ASTD	TAMSFN	Accuracy	Precision	Recall	F1-Score	Kappa
w/o MRNF	✗	✓	✓	0.6250	0.7457	0.6250	0.6465	0.5875
w/o ASTD	✓	✗	✓	0.6501	0.6791	0.6501	0.6501	0.6151
w/o TAMSFN	✓	✓	✗	0.6373	0.6672	0.6373	0.6390	0.6010
w/o MRNF&ASTD&TAMSFN	✗	✗	✗	0.6073	0.7036	0.6073	0.6227	0.5680
AHFN	✓	✓	✓	0.6593	0.6709	0.6593	0.6592	0.6252

**Table 7:** Performance comparison of different architectural design choices on the RML2016.10A dataset.

Model	Replaced Component	Main Modification	Accuracy	Precision	Recall	F1-Score	Kappa
A-CHAIN	Signal Embedding Module	Replace the temporal graph with a chain graph (k=1).	0.6408	0.6824	0.6408	0.6405	0.6048
A-KNN	Signal Embedding Module	Replace the fixed temporal graph with a feature-based KNN graph (K=14).	0.6290	0.6744	0.6290	0.6314	0.5918
A-MLP	KAN-based encoder	Replace KAN with a parameter-matched MLP (hidden size =920).	0.6209	0.7489	0.6209	0.6432	0.5830
A-FIX	Adaptive thresholding	Replace the adaptive threshold with a fixed threshold (τ=0.1).	0.6394	0.6785	0.6394	0.6413	0.6034
A-GCN	CustomGatedGCN	Replace CustomGatedGCN, including GSAA and message modulation, with GCNConv layers	0.6327	0.6693	0.6327	0.6331	0.5959
A-GRU	Mamba	Replace Mamba with a unidirectional GRU.	0.6474	0.6784	0.6474	0.6431	0.6122

**Table 8:** Performance comparison under different three-level TopK ratio settings.

Three-Level TopK Ratio	Accuracy	Precision	Recall	F1-Score	Kappa
[0.2,0.4,0.6]	0.6515	0.6796	0.6515	0.6508	0.6167
[0.3,0.5,0.7]	0.6562	0.6769	0.6562	0.6539	0.6218
[0.4,0.6,0.8]	0.6593	0.6709	0.6593	0.6592	0.6252
[0.5,0.7,0.9]	0.6500	0.6801	0.6500	0.6477	0.6150
[0.6,0.8,0.9]	0.6473	0.6918	0.6473	0.6440	0.6120

**Table 9:** Few-shot classification performance of different models.

Model	Shot	Accuracy	Precision	F1-Score	Kappa	Low-SNR Acc.
AHFN	10	51.91±0.08	59.41±0.85	51.97±0.40	47.10±0.09	31.50±0.01
	50	56.76±0.46	66.83±0.72	58.32±0.43	52.43±0.50	34.38±0.27
	100	58.66±0.45	69.97±1.08	60.51±0.50	54.53±0.50	35.52±0.25
	200	60.72±0.13	72.27±0.65	63.08±0.15	56.79±0.15	36.61±0.07
MCLDNN	10	39.63±2.88	39.97±3.21	37.54±4.47	33.59±3.17	25.86±0.47
	50	51.71±0.48	57.62±0.68	51.27±0.27	46.89±0.53	29.98±0.95
	100	53.76±0.11	62.97±2.00	54.59±0.85	49.14±0.12	31.65±0.20
	200	55.92±0.33	64.22±1.22	56.86±1.02	51.51±0.36	33.76±0.30
TLDNN	10	39.64±1.76	39.67±3.24	36.70±3.73	33.60±1.93	26.30±0.24
	50	50.67±0.33	54.91±0.56	49.70±0.95	45.73±0.36	32.44±0.34
	100	55.67±0.24	62.68±0.79	56.02±0.63	51.23±0.27	34.23±0.26
	200	57.63±0.18	66.56±0.71	58.76±0.33	53.40±0.20	35.63±0.29

**Figure A1:**
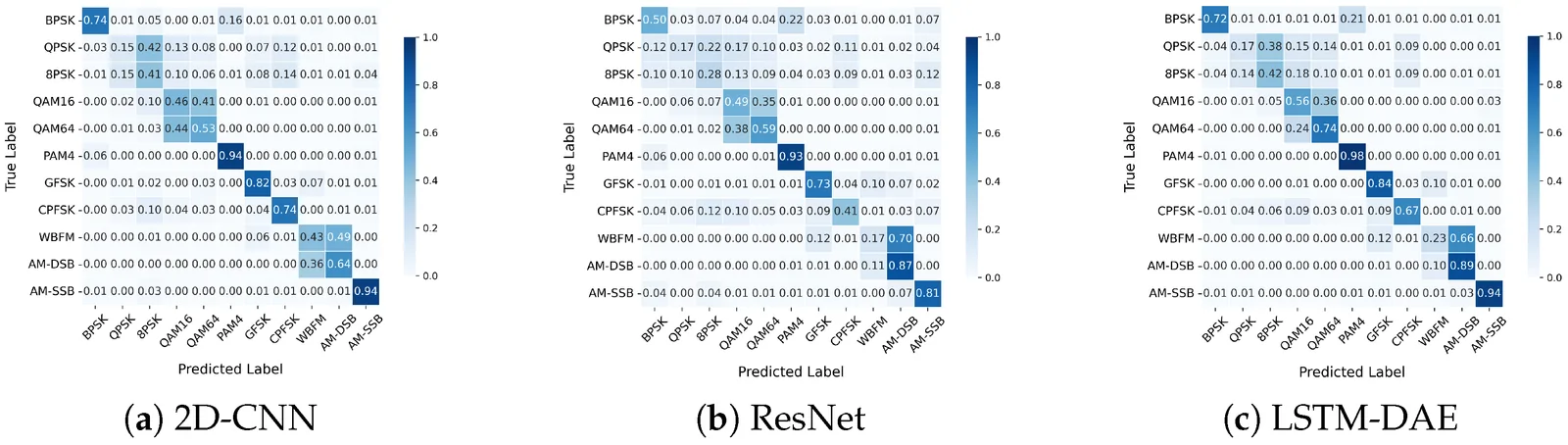

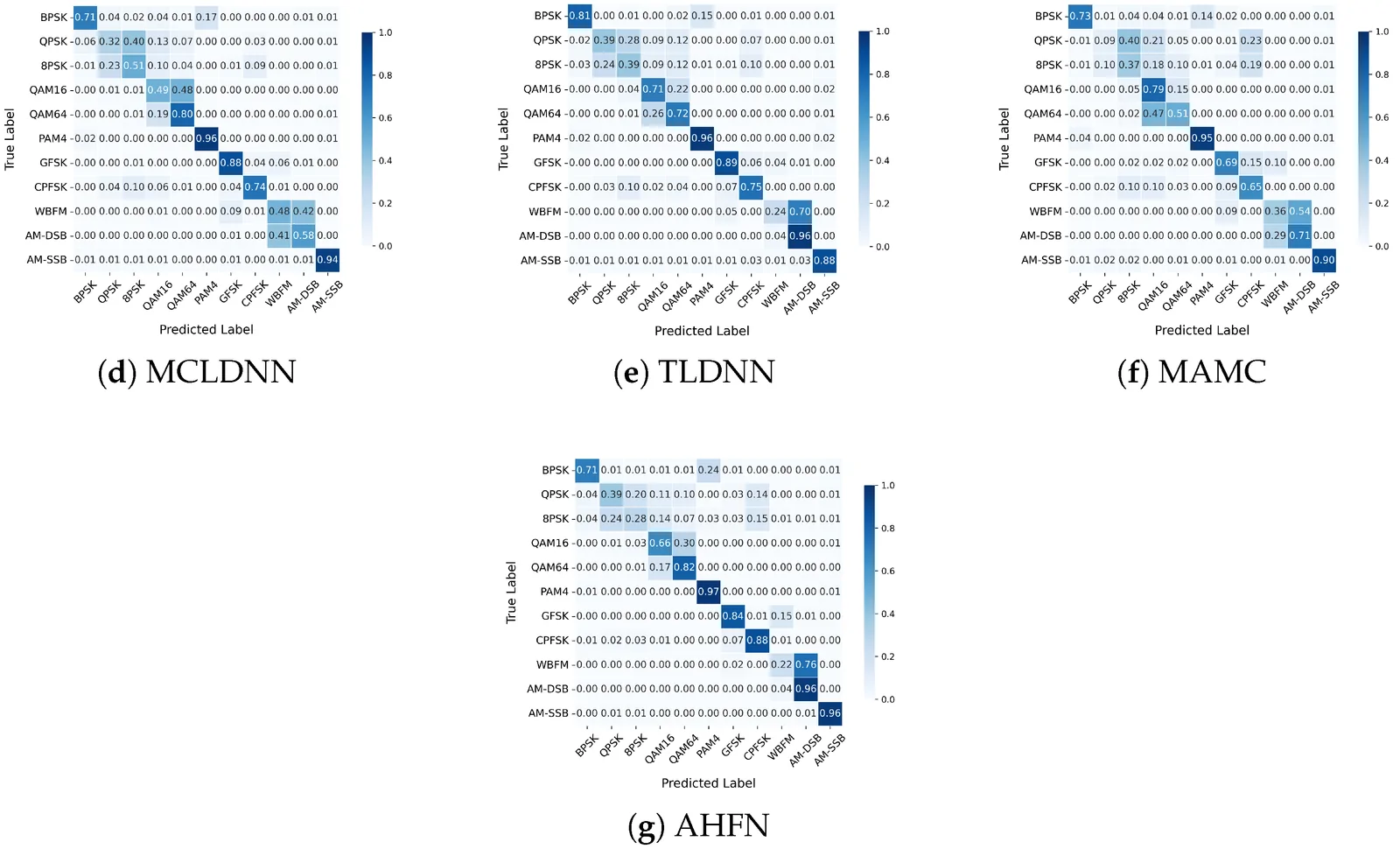
Confusion matrices of different models on RML2016.10A at −4 dB.

**Figure A2:**
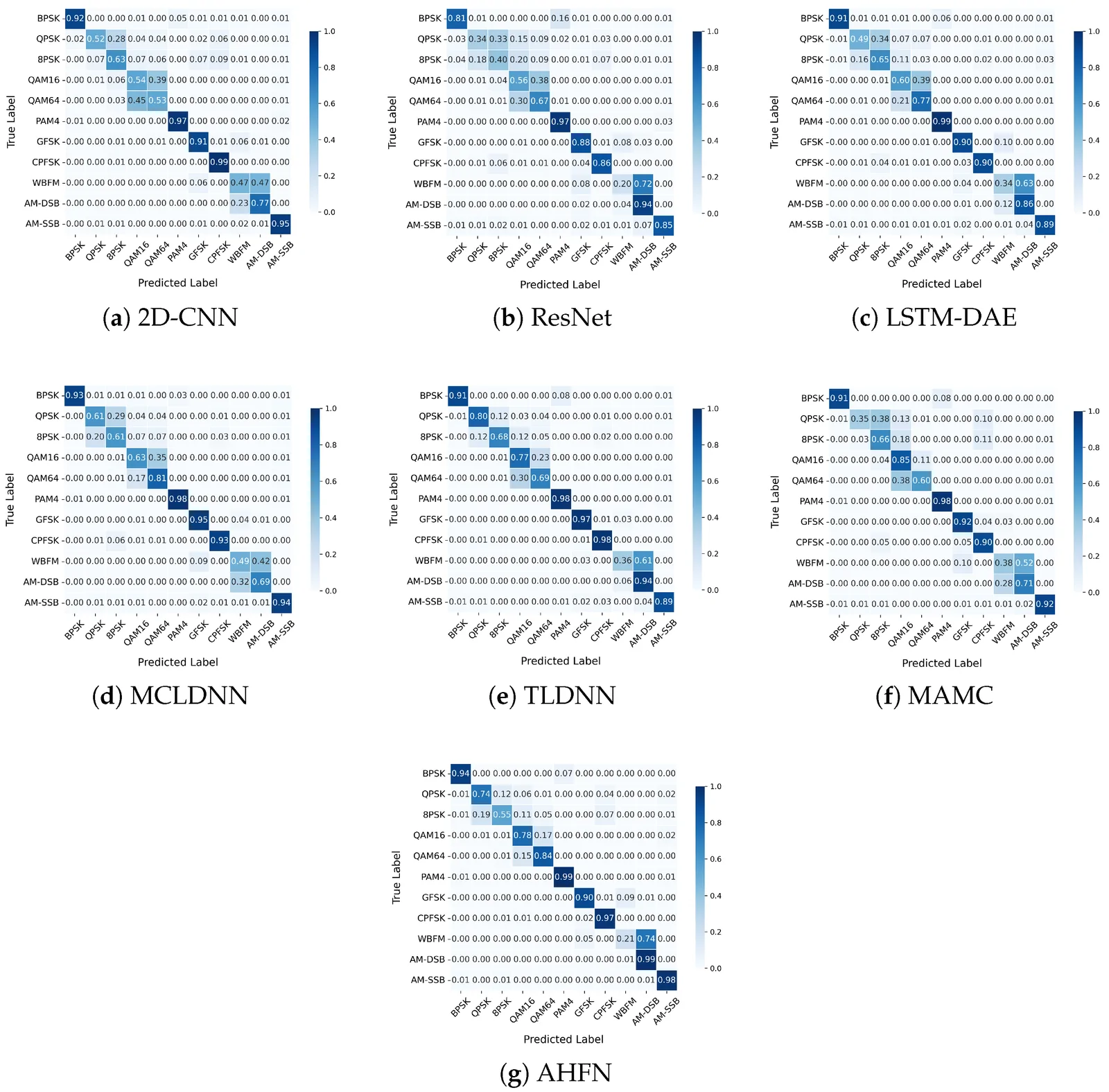
Confusion matrices of different models on RML2016.10A at −2 dB.

## Data Availability

The datasets analyzed during the current study are publicly available. The RadioML 2016.10A and RadioML 2016.10B datasets can be obtained from the DeepSig repository: https://www.deepsig.ai/datasets (accessed on 5 January 2026).

## Appendix A. Additional Confusion Matrices

The additional confusion matrices in Figure A1 and Figure A2 provide a more detailed view of class-wise behavior in the low-SNR transition region. At −4 dB, most baseline models still exhibit evident off-diagonal confusion, especially among modulation types with similar constellation or envelope characteristics, indicating that noise severely weakens class boundaries before the signal structures become sufficiently separable. In contrast, AHFN already shows a clearer diagonal tendency at this SNR, suggesting that the proposed framework can recover part of the discriminative structure even when the received features remain highly fragmented. When the SNR increases to −2 dB, the classification patterns of all models improve, but the improvement of AHFN is more structurally consistent: the diagonal responses become more concentrated, while scattered misclassifications are further reduced. This transition indicates that AHFN does not merely benefit from increased SNR, but better converts the emerging signal structures into stable class-level decisions. Such behavior is consistent with the design of AHFN, where multi-resolution fusion enhances weak modulation cues, adaptive denoising reduces noise-sensitive responses, and topology-aware local–global modeling supports more reliable feature propagation across related signal components.
